# Selecting the Optimal Calculation Method and Chemical Reagents in Surface Energy Tests of Asphalt Materials

**DOI:** 10.3390/ma18122833

**Published:** 2025-06-16

**Authors:** Longchang Niu, Chongzhi Tu, Gongying Ding

**Affiliations:** 1 School of Civil Engineering and Architecture, Wuyi University, Jiangmen 529020, China; m15872368330_1@163.com (L.N.); dgy2517@126.com (G.D.); 2 Guangzhou Expressway Co., Ltd., Guangzhou 510555, China

**Keywords:** asphalt pavement, asphalt surface energy parameter, chemical reagent, total least squares method, least squares method, leap degree

## Abstract

In surface energy tests of asphalt materials, the inaccuracy of the calculation method (e.g., least squares (LS)) and the arbitrary selection of chemical reagent combinations lead to unstable results, threatening the quantitative evaluation of asphalt–aggregate adhesion durability. This study addresses these two scientific deficiencies with the following findings: (1) when simultaneous equations are used to calculate the asphalt surface energy parameters, the total least squares method should be used instead of the classical least squares method to reduce the fitting error; (2) the selection of the reagent combination should be based on which one is the most rational in terms of the physical characterization, leap degree, abnormal values, and other requirements, and the reagent combination with the fewest abnormal values should be chosen as the best scheme. The results show that (1) compared with the classical least squares method, the total least squares method reduces the fitting error between the calculated and real values of asphalt surface energy parameters and improves the accuracy and stability of the calculation results; (2) the best reagent combination scheme is WFSD (distilled water + formamide + dimethyl sulfoxide + diiodomethane). The calculated values of asphalt surface energy parameters were more accurate and reasonable, and the calculation results had no abnormal values. Compared with WFEG (distilled water + formamide + ethylene glycol + glycerol), the error rate of the reagent combination scheme WFSD in calculating the total surface energy of two kinds of asphalt was reduced by 17.71% and 64.80%, respectively. These findings establish a reliable framework for the accurate quantification of surface energy, addressing the critical issue of reagent-dependent variability in the results and strengthening the scientific basis for evaluating the durability of asphalt pavement.

## 1. Introduction

In the design of surface asphalt mixtures, asphalt–aggregate adhesion is a key concern, directly impacting road performance parameters such as fatigue life, self-healing ability, and water stability. Current domestic/foreign specifications commonly use the boiling test to evaluate adhesion; however, this test only provides qualitative grades [1] without quantitative results. The surface energy method determines the binding energy of interface adhesion as a quantitative index, which is closely linked to interface cracking [2,3], and is widely applied to study mixture aging, self-healing, water damage, and other factors during service [4,5,6,7].

The primary prerequisite for the quantitative evaluation of the adhesion mentioned above is to accurately measure the surface energy parameters of the asphalt and aggregate. For this reason, the Good–van Oss–Chaudhury (GvOC) theoretical system has been widely adopted in the road industry; in this system, the asphalt and aggregate have three basic surface energy parameters—the nonpolar component $\gamma^{LW}$, the polar acid component $\gamma^{+}$, and the polar base component $\gamma^{-}$—which together constitute the total surface energy *γ* [8,9,10,11]. For asphalt materials, the usual test methods for measuring surface energy parameters are the Wilhelmy plate method and the sessile drop method. Compared to the sessile drop method, the Wilhelmy plate method provides more stable contact angle data [12,13]. Thus, the Wilhelmy plate method is the preferred method for testing asphalt surface energy and will be applied later in this study.

**Figure 1:**
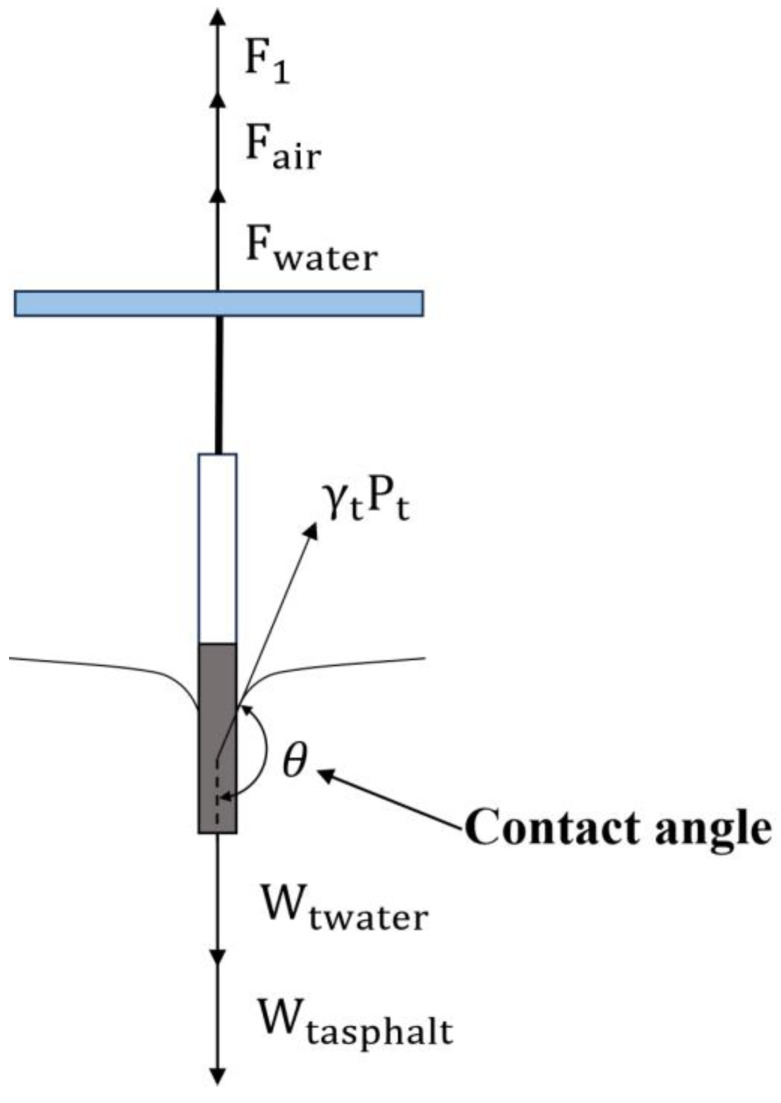
Schematic diagram of contact angle.

To evaluate the asphalt surface energy using the Wilhelmy plate method, it is necessary to select three or more kinds of chemical reagents with known surface energy parameters as a combination of reagents and measure the contact angles *θ* between various chemical reagents and asphalts using tests, as shown in Figure 1.

Next, statically indeterminate equations should be established to determine the surface energy parameters of asphalt using the least squares (LS) method [14]. The widespread prevalence of LS can be attributed to its straightforward computational framework and well-established theoretical underpinnings. As a cornerstone of linear regression analysis, LS finds extensive application in diverse fields, such as data modeling and trend forecasting. In the context of recent research on calculating asphalt surface energy, for instance, LS continues to be the method of choice, as evidenced by previous studies [3,4,15].

The surface energy parameters of known chemical reagents must be determined before solving the equations. In most studies, the surface energy parameters of various chemical reagents listed in the literature are often directly selected. Through the analysis of extensive data and long-term experimental research, it has been found that the surface energy parameters of the same asphalt measured with different reagent combinations are obviously different, and for some reagent combinations, the surface energy parameters cannot even be calculated [11,16,17]. The negative square root often appears in the solution process, which is contrary to the physical implication of asphalt surface energy. Some researchers believe that the negative square root is caused by ill-conditioned equations and try to solve it using singular value decomposition [18], but this problem persists; others directly set the surface energy parameter value of the negative square root to zero [19], which is inconsistent with the basic properties of asphalt.

At the same time, many studies show that the asphalt surface energy parameters calculated with different reagent combinations are considerably different. When selecting reagent combinations, some scholars treat the surface energy parameters of reagent combinations as a coefficient matrix and then calculate the condition number to compare the stability of asphalt surface energy parameters calculated with different reagent combinations [20,21]. However, when too many kinds of reagents are involved, a feasible reagent selection scheme cannot be determined by the condition number. Some scholars plot the curve of the relationship between the total surface energy $\gamma_{L}$ of the chemical reagents and the value of $\gamma_{L}cos\theta$ and select reagent combinations according to the smoothness of the fitting line [22,23]. Nonetheless, even using this selection method, the calculated values of asphalt surface energy parameters still have large differences.

Therefore, based on the two perspectives of optimizing the equation calculation method and reagent combination, the main reasons for the differences between asphalt surface energy parameters measured using the Wilhelmy plate method can be summarized as follows:

(1) When the LS method is used to solve asphalt surface energy parameters, the optimal fitting method is not used, which causes a large fitting error between the calculated and real values of asphalt surface energy parameters;
(2) There is a lack of a reasonable and effective evaluation method for selecting different reagent combinations.

**Figure 2:**
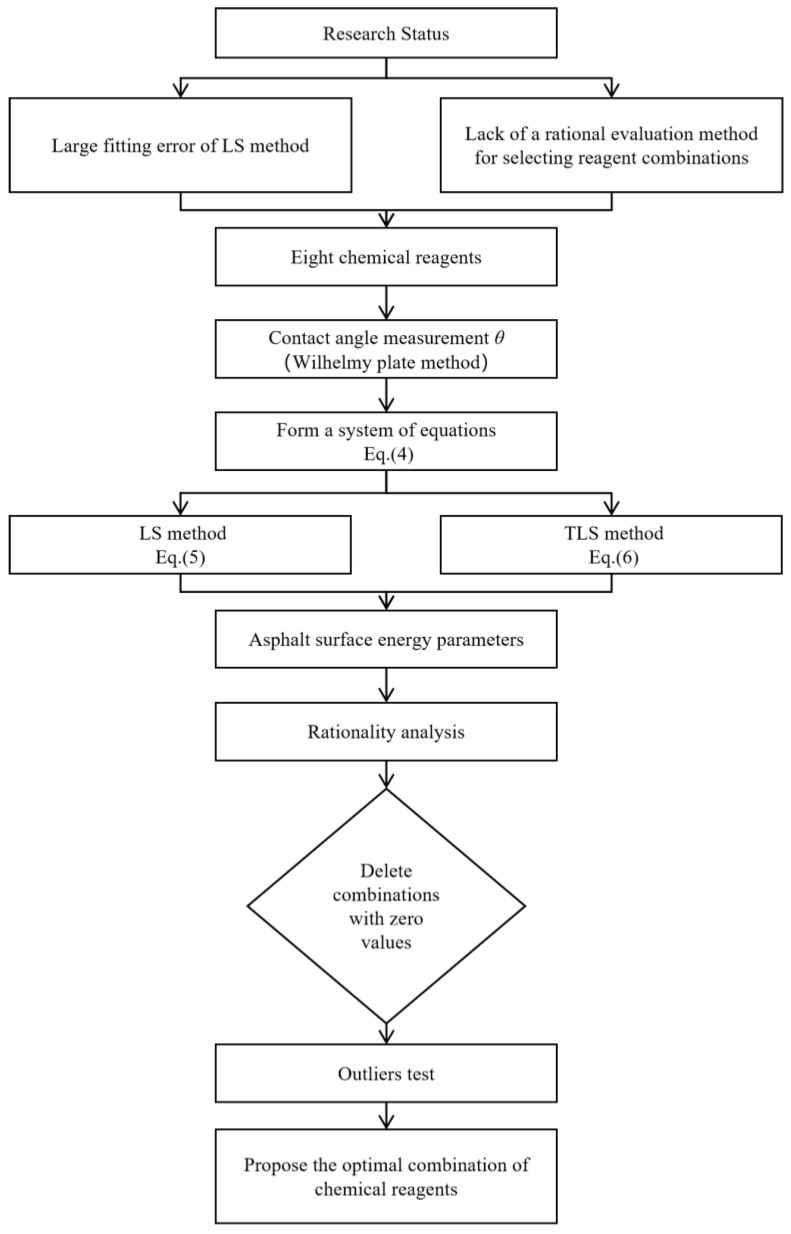
The methodology flowchart.

Therefore, although the surface energy is widely used to evaluate the adhesion of asphalt, problems remain in terms of reagent selection and the calculation method for determining asphalt surface energy, and there is still a lack of research in this area. In order to solve these problems, this study focused on the optimization of the calculation method and the selection of chemical reagents in surface energy tests of road asphalt materials. First, eight chemical reagents were selected to test their contact angles with asphalts using the Wilhelmy plate method. Second, by analyzing the basic principles and geometric interpretation of the LS method in the solution process, the main reasons for the errors were explored, and the total least squares (TLS) method was proposed to calculate asphalt surface energy parameters. Third, the best reagent combination was selected by using the leap degree method. Fourth and last, the main research results are summarized. The methodology flowchart is shown in Figure 2.

## 2. Contact Angle Test

### 2.1. Reagent Selection

In this study, eight chemical reagents were preliminarily selected: distilled water (W), formamide (F), ethylene glycol (E), glycerol (G), dimethyl sulfoxide (S), diiodomethane (D), benzyl alcohol (B), and n-octanol (N). The basic selection principles were as follows [6]:

- The chemical reagent is a single, homogeneous, and pure liquid reagent and does not dissolve or react with asphalt material.
- In order to solve asphalt surface energy parameters using simultaneous equations, the surface energy parameters of chemical reagents must be known;
- Chemical reagent droplets can form a stable contact angle on the surface of asphalt film slides; that is, the total surface energy of the chemical reagent is greater than that of asphalt material.

The above eight chemical reagents were selected because they were easily accessible in the laboratory and are consistent with the basic principles. The polarity parameters of the eight reagents cover a wide range, from highly polar to nonpolar, effectively constraining the surface energy parameters of asphalt. The abbreviations and surface energy parameters of all reagents are listed in Table 1, where W, F, E, G, S, and B are nonpolar reagents, while D and N are unipolar reagents.

The polarity parameters of the eight reagents cover a wide range, from highly polar to nonpolar (as shown in Table 1), effectively constraining the surface energy parameters of asphalt:

- Highly polar reagents (such as water and formamide): They dominate the polar interactions and sensitively reflect the polar components ($\gamma^{+}$ and $\gamma^{-}$) of asphalt.
- Moderately polar reagents (such as ethylene glycol and glycerol): They balance the polar and nonpolar interactions and are used to adjust the condition number of the equation set.
- Weakly polar/nonpolar reagents (such as diiodomethane and n-octanol): They mainly contribute nonpolar dispersion forces and constrain the $\gamma^{LW}$ component of asphalt.

### 2.2. Asphalt Preparation

Two types of asphalt materials, namely, 70# matrix asphalt and SBS (I-D type)-modified asphalt, were selected for this study, and the basic characteristics of each are presented in Table 2. The test results all meet the technical requirements for asphalt pavement construction. Measurements were carried out with reference to the standards for asphalt testing, specifically JTG E20-2011 in China [24].

### 2.3. Measurement Method

Although the Wilhelmy plate method produces more stable results than the sessile drop method, contact angle measurements may still be affected by factors such as asphalt film flatness, reagent droplet acceleration, and instrument accuracy. Therefore, in order to reduce errors caused by the experiment, experimentation was performed in accordance with ASTM D1331-14 [25].

The equipment used in the test was an automatic surface tension meter. Five parallel tests were conducted for each chemical reagent as well as for asphalt, and the average value of three test measurements was calculated to ensure that the test error is within ±2°. The measured results are summarized in Table 3.

## 3. Calculation of Asphalt Surface Energy Parameters

### 3.1. Calculation Principle of Asphalt Surface Energy Parameters

After the contact angles between chemical reagents and asphalts are measured, the asphalt surface energy parameters can be calculated according to the Young–Dupre equation, as shown in Formula (1):

(1) $$\sqrt{\gamma_{S}^{-}\gamma_{L}^{+}}+\sqrt{\gamma_{S}^{+}\gamma_{L}^{-}}+\sqrt{\gamma_{S}^{LW}\gamma_{L}^{LW}}=\frac{1}{2}(1+\cos\theta )\gamma_{L}$$

where $\gamma_{L}^{+}$, $\gamma_{L}^{-}$, $\gamma_{L}^{LW}$, and $\gamma_{L}$ are the three surface energy parameters and total surface energy of the selected chemical reagent, respectively (mJ/m^2^); $\gamma_{s}^{+}$, $\gamma_{\bar{S}}$, and $\gamma_{S}^{LW}$ are the three surface energy parameters of asphalt, respectively (mJ/m^2^); and *θ* is the contact angle measured using the Wilhelmy plate method (°).

Next, the surface energy polar component $\gamma_{s}^{AB}$ and total surface energy $\gamma_{S}$ of asphalt are calculated using the following formulas:

(2) $$\gamma_{s}^{AB}=2\sqrt{\gamma_{s}^{+}\gamma_{s}^{-}}$$

(3) $$\gamma_{s}=\gamma_{s}^{AB}+\gamma_{s}^{LW}$$

As mentioned above, it is imperative to select at least three chemical reagents with known surface energy parameters as reagent combinations to calculate the asphalt surface energy parameters according to the Young–Dupre equation. After contact angles are measured, statically indeterminate equations are established and solved, as shown in Formula (4):

(4) $$\left(\begin{array}{c} \frac{\gamma_{L1}\left(1+\cos\theta_{1}\right)}{2}\\ \frac{\gamma_{L2}\left(1+\cos\theta_{2}\right)}{2}\\ \vdots \\ \frac{\gamma_{Ln}\left(1+\cos\theta_{n}\right)}{2} \end{array}\right)=\left(\begin{array}{ccc} \sqrt{\gamma_{L1}^{LW}} & \sqrt{\gamma_{L1}^{-}} & \sqrt{\gamma_{L1}^{+}}\\ \sqrt{\gamma_{L2}^{LW}} & \sqrt{\gamma_{L2}^{-}} & \sqrt{\gamma_{L2}^{+}}\\ \vdots & \vdots & \vdots \\ \sqrt{\gamma_{Ln}^{LW}} & \sqrt{\gamma_{Ln}^{-}} & \sqrt{\gamma_{Ln}^{+}} \end{array}\right)\left(\begin{array}{c} \sqrt{\gamma_{s}^{LW}}\\ \sqrt{\gamma_{s}^{+}}\\ \sqrt{\gamma_{s}^{-}} \end{array}\right)$$

where $\gamma_{Ln}^{+}$, $\gamma_{Ln}^{-}$, $\gamma_{Ln}^{LW}$, and $\gamma_{Ln}$ are the three surface energy parameters and total surface energy of the $n^{th}$ chemical reagent, respectively (mJ/m^2^), and n≤8; $\theta_{n}$ is the value of the contact angle between the $n^{th}$ chemical reagent and asphalt (°); and $\gamma_{s}^{+}$, $\gamma_{s}^{-}$, and $\gamma_{S}^{LW}$ are the same as in Formula (1).

**Figure 3:**
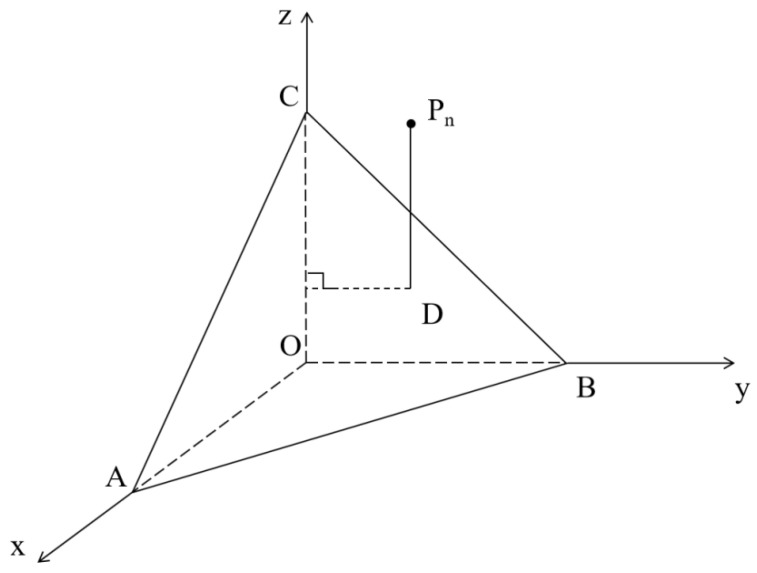
The fitting principle of LS.

In the surface energy test of asphalt materials, Equation (4) is usually solved using the LS method, whose basic principle is to solve and optimize a linear regression equation according to the LS criterion [26,27]. In Formula (4), there are three unknown quantities: $\gamma_{s}^{+}$, $\gamma_{s}^{-}$, and $\gamma_{S}^{LW}$. Consequently, asphalt surface energy parameters are calculated by solving ternary linear equations. The surface energy parameters of different chemical reagents are plotted in a three-dimensional coordinate system and expressed as points P_1_ ($\sqrt{\gamma_{L1}^{LW}}$, $\sqrt{\gamma_{L1}^{-}}$, $\sqrt{\gamma_{L1}^{+}}$), P_2_ ($\sqrt{\gamma_{L2}^{LW}}$, $\sqrt{\gamma_{L2}^{-}}$, $\sqrt{\gamma_{L1}^{+}}$), …, P_n_ ($\sqrt{\gamma_{Ln}^{LW}}$, $\sqrt{\gamma_{Ln}^{-}}$, $\sqrt{\gamma_{Ln}^{+}}$), and n≤8. The plane ABC is obtained by fitting according to LS, which minimizes the average value of the sum of distances P_n_D from all the above points, P_1_, P_2_, …, P_n_, to the plane ABC along the *Z*-axis, as shown in Figure 3.

Thus, the length of the fitting distance P_n_D from any point P_n_ to the plane ABC can be expressed as

(5) $$P_{n}D=\frac{1}{2n}\sum_{1}^{n}\left|\left(2\sqrt{\gamma_{S}^{LW}\gamma_{Ln}^{LW}}+\sqrt{\gamma_{S}^{+}\gamma_{Ln}^{-}}+\sqrt{\gamma_{S}}\gamma_{Ln}^{+}\right)-\gamma_{Ln}(1+\cos\theta_{n})\right|$$

**Figure 4:**
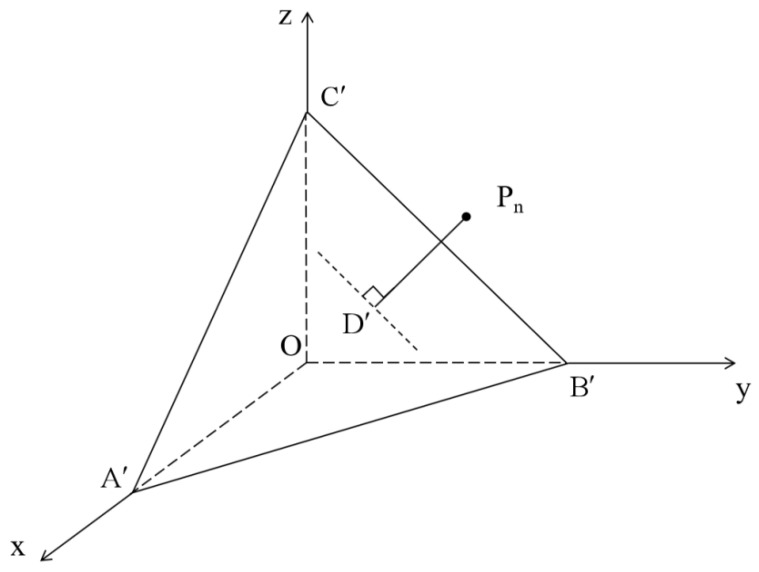
The fitting principle of TLS.

However, in the actual process of measurement, the errors in the raw data are not limited to a single coordinate axis, so LS cannot obtain the shortest fitting distance. As a comparison, using the TLS method to solve Equation (4) can account for all the errors in the direction of all axes [28,29]. Based on the fitting principle of LS, the plane $A^{\prime }B^{\prime }C^{\prime }$ is obtained by fitting according to TLS, which minimizes the average value of the sum of the vertical distances $P_{n}D^{\prime }$ from all the above points to the plane $A^{\prime }B^{\prime }C^{\prime }$, as shown in Figure 4.

Likewise, the length of the fitting distance $P_{n}D^{\prime }$ from any point P_n_ to the plane $A^{\prime }B^{\prime }C^{\prime }$ can be expressed as

(6) $${P_{n}D}^{\prime }=\frac{1}{n}\sum_{1}^{n}\left|\frac{2\left(\sqrt{\gamma_{S}^{LW}}\gamma_{Ln}^{LW}+\sqrt{\gamma_{S}^{+}}\gamma_{Ln}^{-}+\sqrt{\gamma_{S}^{-}}\gamma_{Ln}^{+}\right)-\gamma_{Ln}(1+\cos\theta_{n})}{2\sqrt{{\left(\sqrt{\gamma_{Ln}^{LW}}\right)}^{2}+{\left(\sqrt{\gamma_{Ln}^{-}}\right)}^{2}+{\left(\sqrt{\gamma_{Ln}^{+}}\right)}^{2}}}\right|$$

By comparison, it can be seen that $P_{n}D^{\prime }$ < $P_{n}D$; that is, TLS can be used to determine the plane with smaller fitting errors to calculate more accurate asphalt surface energy parameters.

### 3.2. Calculation of Asphalt Surface Energy Parameters Using TLS

**Figure 5:**
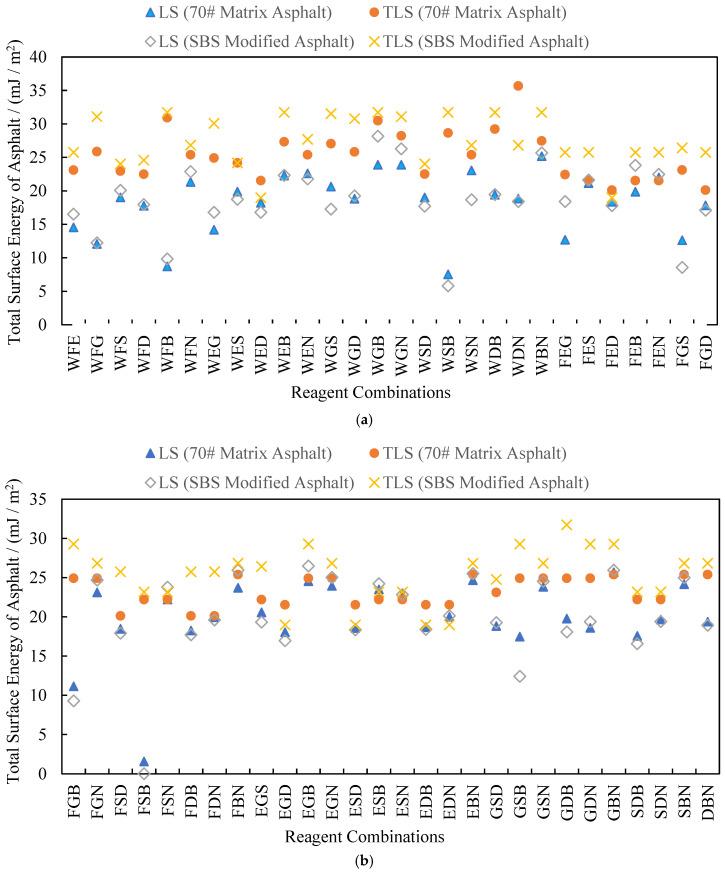
Total surface energy of asphalts calculated for combinations of three chemical reagents. (**a**) Part One; (**b**) Part Two.

**Figure 6:**
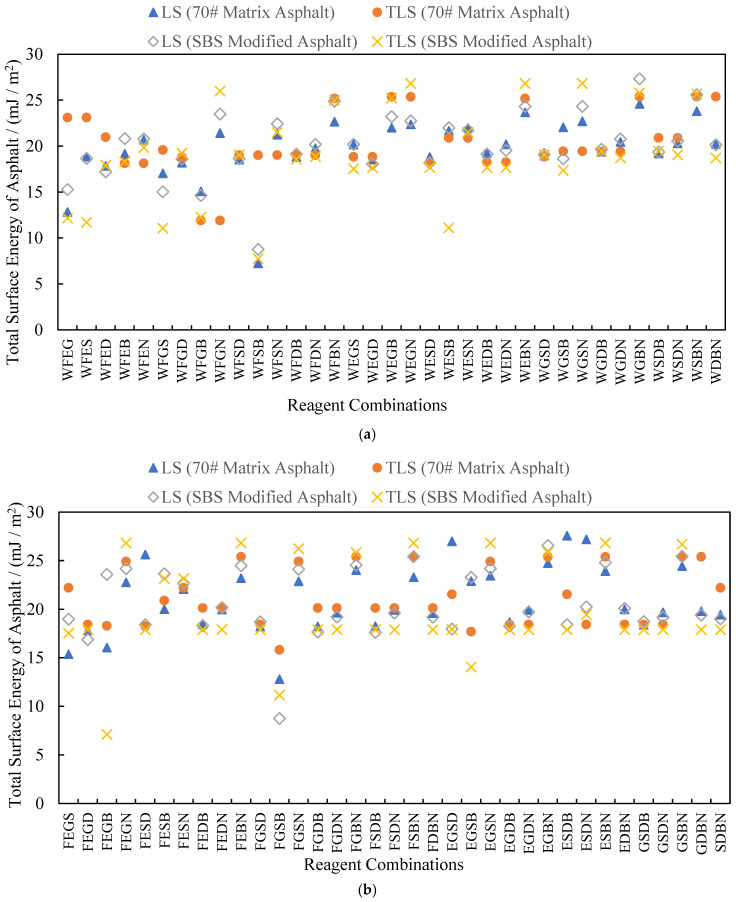
Total surface energy of asphalts calculated for combinations of four chemical reagents. (**a**) Part One; (**b**) Part Two.

At least three chemical reagents are needed to calculate asphalt surface energy parameters using TLS. If three of the eight chemical reagents with known surface energy parameters are randomly selected to solve Equation (4), then there are $C_{8}^{3}=56$ reagent combinations; if four reagents are selected, then there are $C_{8}^{4}=70$ reagent combinations. Among combinations, WFE represents the reagent combination of distilled water + formamide + ethylene glycol, while WFEG represents the reagent combination of distilled water + formamide + ethylene glycol + glycerol, and so on. For each reagent combination, Microsoft Excel was used to calculate the asphalt surface energy parameters by using LS and TLS, respectively. All calculated values of the total surface energy of asphalt $\gamma_{S}$ are summarized in Figure 5 and Figure 6.

According to the analysis of the data in the figures above for 70# matrix asphalt and SBS-modified asphalt, when the LS and TLS methods are used for calculations, the absolute values of the total average change rate of surface energy are 35.1% and 235.0%, indicating a significant distinction between the calculation results of the two methods. When the reagent combination is FSB, the maximum absolute values of the change rate are as high as 1316.2% and 23,060.0%. This shows that the data processing method has a significant impact on the results.

The surface energy of asphalt is a basic physical parameter that reflects its adhesion, and it will not change with different reagent combinations. However, in practical calculations, the total surface energy of asphalt calculated with different reagent combinations will still have large differences. Using the LS method, the coefficients of variation of total surface energy for the 70# matrix asphalt and SBS-modified asphalt are 20.32% and 26.35%, respectively; using the TLS method, the coefficients of variation of the total surface energy of the two asphalts are 15.92% and 24.65%, respectively. Compared to LS, the coefficient of variation of the calculated results of TLS is smaller, so the results calculated with the TLS method are more stable. The LS method only minimizes the single-axis (*Z*-axis) error, while the TLS method minimizes the full-dimensional vertical error, especially in ill-conditioned equations (for example, the change rate of the calculated values for the FSB combination is reduced from 23,060% to an acceptable range).

At the same time, if the total surface energy of the two kinds of asphalt is calculated using the TLS method, the coefficients of variation of the total surface energy of the 70# matrix asphalt with three- and four-reagent combinations are 14.22% and 12.57%, respectively, while the coefficients of variation of the total surface energy of SBS-modified asphalt are 24.77% and 14.15%, respectively. Undoubtedly, the total surface energy of asphalt calculated with four-reagent combinations is more stable. All conclusions above indicate that the selection of reagents will directly affect the accuracy and stability of results in surface energy tests of asphalt materials. Therefore, the following paragraphs concentrate on the analysis of the calculated results and the optimization of reagent combinations.

### 3.3. Analysis of Test Results

The analysis of surface energy tests for 70# asphalt and SBS-modified asphalt reveals significant differences between calculation methods. The total least squares (TLS) method outperforms the least squares (LS) method in reducing fitting errors and improving stability, as it considers full-dimensional errors rather than single-axis errors. The selected reagent combination also impacts the results: four-reagent combinations generally yield more stable surface energy parameter calculations than three-reagent ones, likely due to the redundancy of hyperstatic equations. This study highlights the importance of rational reagent selection (including nonpolar and polar reagents) and appropriate calculation methods to ensure accuracy in asphalt surface energy testing. Further optimization of reagent combinations and the validation of calculation methods are recommended for reliable adhesion performance evaluation.

## 4. Optimization of Reagent Combinations

In this study, the rationality and outliers of the calculated results were analyzed to determine the optimal reagent combination scheme.

### 4.1. Rationality Analysis of Calculated Results

Through observing the values of every component of asphalt surface energy calculated using the TLS method described above, it was revealed that calculations with some reagent combinations result in zero values for certain surface energy components of asphalt, as shown in Table 4.

**Figure 7:**
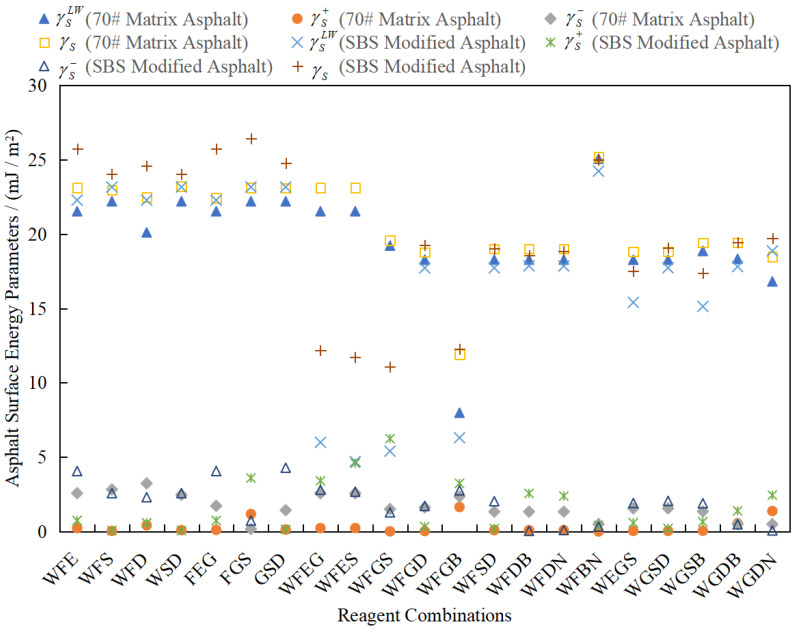
The calculated values of asphalt surface energy parameters meeting the requirements.

As a complex mixture composed of various polar/nonpolar organic compounds, asphalt exhibits both dispersion forces (nonpolar interactions) and polar interactions (such as hydrogen bonding and dipole interactions) between molecules. Therefore, its surface energy must include nonzero polar and nonpolar components. It is impossible for asphalt to be a unipolar or nonpolar substance, and its three surface energy components should be greater than zero. By excluding reagent combinations with zero values in the calculation results (WFN, FED, etc.; see Table 4), the 21 effective combinations selected (Figure 7) all meet the condition of three nonzero components, and the proportion of γL in the total surface energy in the calculation results is over 80%, which conforms to the physical characteristics of asphalt with nonpolar interactions as the main component. Thus, the calculated results of the above reagent combinations violate the basic chemical properties of asphalt. Furthermore, in the process of optimization, these reagent combination schemes should be excluded first, and only the reagent combinations whose calculated surface energy components are not zero should be considered.

In addition, according to the calculation principle of the Young–Dupre equation, droplets of the selected chemical reagents should be able to form a stable contact angle on the surface of asphalt slides during the test [8]. If the calculated value of the total surface energy of asphalt is greater than that of the chemical reagent, then the reagent cannot form a stable contact angle on the surface of the asphalt slide, which is inconsistent with the calculation principle of the Young–Dupre equation. Consequently, it is necessary to exclude such reagent combination schemes. A total of 18 reagent combinations meet the above two criteria, namely, WFB, WFG, WGS, WGD, WGB, WGN, WSB, WDB, WDN, WEG, WEB, WBN, FGB, EGB, GSB, GDB, GDN, and GBN.

In addition to the above 18 reagent combinations, the remaining 21 reagent combinations and the calculated values of asphalt surface energy parameters are summarized in Figure 7.

As can be seen in Figure 7, the distribution ranges of calculated surface energy parameters of the two kinds of asphalt are as follows: 17 mJ/m^2^ ≤ $\gamma_{S}^{LW}$; $\gamma_{S}^{LW}$ ≤ 25 mJ/m^2^; 0 mJ/m^2^ ≤ $\gamma_{S}^{-}$; and $\gamma_{s}^{+}$ ≤ 3 mJ/m^2^. Moreover, most $\gamma_{S}^{LW}$ values are much greater than $\gamma_{S}^{-}$ and $\gamma_{s}^{+}$ and are close to $\gamma_{S}$, indicating that asphalt is a substance dominated by intermolecular nonpolar forces, which is consistent with the basic chemical properties of asphalt.

Meanwhile, comparing different reagent combinations that meet the requirements shows that some differences in the calculated values of asphalt surface energy parameters remain.

Therefore, in order to obtain more accurate and reasonable asphalt surface energy parameters, it is crucial to establish a quick and effective method for selecting reagent combinations.

### 4.2. Testing for Outliers in the Calculated Results

In the calculated results that meet the requirements for surface energy parameters of the same kind of asphalt, the differences between most of the data points are tiny; therefore, it can be assumed that the main factor leading to differences is the test error caused by different reagent combinations. However, a small number of data points are significantly different from other data and can be called outliers [30,31,32,33]. In order to further eliminate the interference of outliers in the raw data and ensure the accuracy and reliability of the calculated results, it was necessary to conduct an outlier test on the calculated results of the 21 reagent combinations in Figure 7.

The basic steps of the outlier test are as follows: the calculated results for surface energy parameters of a certain asphalt with 21 reagent combinations are arranged in order from small to large and set as $X_{\left(1\right)},X_{\left(2\right)},\ldots ,X_{\left(n-1\right)},X_{\left(n\right)},n\leq 21$. $\mu_{k}$ is a point estimate of the expectation *μ* that depends only on the sequence $X_{\left(1\right)},\ldots ,X_{\left(k\right)}$, and $\frac{\mu_{k+1}}{\mu_{k}}$ is called the leap degree of expectation *μ* at a point *k*, or the leap degree at point *k* for short. The calculation formula for the leap degree is as follows:

(7) $$\mu_{k}=\frac{\sum_{i=1}^{k}x_{\left(i\right)}+(n-k)x_{\left(k\right)}}{k}$$

(8) $$\mu_{k+1}=\frac{\sum_{i=1}^{k+1}x_{\left(i\right)}+\left[n-(k+1)\right]x_{\left(k+1\right)}}{k+1}$$

(9) $$D_{k}=\frac{\mu_{k+1}}{\mu_{k}}$$

where $\mu_{k}$ and $\mu_{k+1}$ are both point estimates of the expectation *μ*; *k* is a sequence of calculated values for any of the asphalt surface energy parameters arranged in order, k=1,2,3,…,n; and $D_{k}$ is the leap degree at point *k*.

Thus, the existence of an outlier will certainly cause an intermittent leap in the point estimate of the expectation, and when more than one outlier exists, the maximum leap point of the point estimate of the expectation—namely, the point with the largest leap degree—is most likely to be the starting point of abnormal data [32]. After calculating the leap degree at every point using Equations (7)–(9), in order from left to right, when the first maximum value greater than one appears in a leap degree, the point corresponding to that leap degree is the end point of small outliers; that is, all the calculated values before that point (including that point) are small outliers. In order from right to left, when the first maximum value greater than one appears in a leap degree, the point corresponding to that leap degree is the starting point of large outliers; that is, all the calculated values after that point (excluding that point) are large outliers.

There are three types of cases in which a set of data has outliers—there are only small outliers; there are only large outliers; or there are both small and large outliers [29]. The reagent combinations and leap degrees corresponding to every point are provided in Appendix A Table A1 and were obtained according to the above test steps, and the numbers of outliers in the calculated results of the 21 reagent combinations are listed in Appendix A Table A2. In Table A1, the reagent combinations shaded gray are those with outliers in the calculated results.

As can be seen from Appendix A Table A1 and Table A2, for 70# matrix asphalt and SBS-modified asphalt, the number of outliers in the calculated results of the 21 reagent combinations varies from 0 to 4, with the lowest number (0) found for the reagent combination WFSD—that is, there are no outliers. Therefore, through the leap degree test, the best reagent combination scheme is finally determined to be WFSD.

### 4.3. Analysis of Results

In this study, the optimal reagent combination scheme was determined through the following three steps:

(1) Based on three basic principles, eight chemical reagents with known surface energy parameters were preliminarily selected: W, F, E, G, S, D, B, and N. Then, 126 reagent combinations were formed randomly.
(2) After the contact angles were measured using the Wilhelmy plate method and the asphalt surface energy parameters were determined using TLS, the rationality of the calculated results for all reagent combinations was analyzed, and those that were obviously unreasonable were eliminated. Finally, 21 reagent combinations that met the requirements were identified.
(3) Through the leap degree test, the reagent combination scheme with the fewest outliers was selected from the 21 reagent combinations so as to achieve the research purpose of this study.

Through a series of laboratory tests, theoretical calculations, and data analyses combined, the combination scheme WFSD was selected, as there are no outliers in the calculated results of asphalt surface energy parameters with this combination, and the calculated results are closer to the real values. The calculated results of asphalt surface energy parameters with WFEG, the most commonly used reagent combination, are summarized in Table 5. According to the comparative analysis, for 70# matrix asphalt and SBS-modified asphalt, the error rate with the optimal reagent combination scheme, WFSD, in calculating the total surface energy of asphalt, $\gamma_{S}$, is reduced by 17.71% and 64.80%, respectively. Stability reflects the degree of dispersion, and it is necessary to use the homogeneity-of-variance test (*F*-test) to test the stability of the results of the two experimental methods. After calculation, the variance in the TLS group was significantly lower than that in the LS group (F(4,4) = 6.39, *p* = 0.006 < 0.05), indicating that TLS produces more stable results in calculating asphalt surface energy.

## 5. Conclusions

The main conclusions of this paper are as follows:

(1) Compared to the LS method, the TLS method is more suitable for solving the linear equations established using the Young–Dupre equation. The TLS approach reduces the fitting error between the calculated and real values of asphalt surface energy parameters and improves the accuracy and stability of the calculated results. The coefficients of variation of the total surface energy of the 70# matrix asphalt and SBS-modified asphalt calculated using TLS are 15.92% and 24.65%, respectively, which are 21.65% and 6.45% reductions compared to those obtained with the LS method. The LS method only minimizes the single-axis (*Z*-axis) error, while the TLS method minimizes the full-dimensional vertical error, especially in ill-conditioned equations (for example, the change rate of the calculated values for the FSB combination is reduced from 23,060% to an acceptable range).
(2) In this study, the number of outliers in the calculated results was determined by analyzing the rationality of the calculated results and using the leap degree test method. Finally, among 126 reagent combinations, the optimal reagent combination scheme was determined to be WFSD; there were no outliers in the calculated results of asphalt surface energy parameters, which were closer to the real values. For the 70# matrix asphalt and SBS-modified asphalt, compared to the most commonly used reagent combination, WFEG, the error rate of the reagent combination WFSD in calculating the total surface energy of asphalt was reduced by 17.71% and 64.80%, respectively.
(3) The leap degree test method can quickly and accurately determine the reagent combination with the fewest outliers, and the selected resolution is more reasonable and reliable. Therefore, it can be used to select the optimal reagent combination. These research results can provide an experimental basis for the accurate calculation of asphalt surface energy parameters and assist in accurately analyzing the performance of adhesion between asphalt and aggregate. Thus, surface energy tests for more kinds of asphalt, asphalt binders, and aggregates will be conducted in the future, and the optimal reagent combination scheme suitable for surface energy tests of different road materials will be explored based on classification.

Through the optimization of calculation methods and reagent combinations, a more accurate framework for testing the surface energy of asphalt was established, which provides an experimental basis for the quantitative evaluation of the adhesion between asphalt and aggregate. In the future, this approach can be further extended to analyze more asphalt types (such as mortar and modified asphalt) and aggregate surface energy tests, explore the optimal reagent combination of different road materials based on classification, and improve the application of surface energy theory in road engineering.

## Figures and Tables

**Table 1:** Abbreviations for chemical reagents and their surface energy parameters.

Chemical Reagent (Abbreviation)	Surface Energy Parameter/(mJ/m^2^)				
	$\gamma^{LW}$	$\gamma^{AB}$	$\gamma^{+}$	$\gamma^{-}$	$\gamma_{L}$
Distilled Water (W)	21.80	51.00	25.50	25.50	72.80
Formamide (F)	39.00	19.00	2.28	39.60	58.00
Ethylene Glycol (E)	29.00	19.00	3.00	30.10	48.00
Glycerol (G)	34.00	30.00	3.92	57.40	64.00
Dimethyl Sulfoxide (S)	36.00	8.00	0.50	32.00	44.00
Diiodomethane (D)	50.80	0	0.01	0	50.80
Benzyl Alcohol (B)	28.60	11.40	0.95	34.20	40.00
N-Octanol (N)	27.50	0	0	3.97	27.50

**Table 2:** Properties of asphalt materials.

Property				Asphalt Material		Technical Requirement	
				70# Matrix Asphalt	SBS (I-D Type)-Modified Asphalt	70# Matrix Asphalt	SBS (I-D Type)-Modified Asphalt
Penetration at 25 °C, 0.1 mm				64.0	53.0	60.0–70.0	40.0–60.0
Softening Point (T_R&B_), °C				47.0	66.0	≥46.0	≥60.0
Ductility, cm		10 °C		35.0	/	≥20.0	/
		5 °C		/	41.0	/	≥20.0
Dynamic Viscosity at 60 °C, (Pa·s)				208.4	/	≥180.0	/
Kinematic Viscosity at 135 °C, (Pa·s)				/	2.8	/	≤3.0
Wax Content (Distillation Method),%				1.6	/	≤2.2	/
Flash Point, °C				295.0	255.0	≥260.0	≥230.0
Solubility,%				99.8	99.4	≥99.5	≥99.0
Density, (g/cm^3^)				1.04	1.07	/	/
Residue after RTFOT	Quality Loss			−0.07	−0.12	≤±0.80	≤±1.00
	Penetration Ratio at 25 °C,%			67.0	75.0	≥61.0	≥65.0
	Ductility, cm		10 °C	11.0	/	≥6.0	/
			5 °C	/	22.0	/	≥15.0

**Table 3:** Contact angle data (°).

Test Method	Asphalt Material	Chemical Reagent							
		W	F	E	G	S	D	B	N
Wilhelmy Plate Method	70# Matrix Asphalt	104.33	91.96	87.64	95.20	73.44	78.21	66.54	22.84
	SBS-Modified Asphalt	101.43	89.06	91.30	90.81	71.79	79.23	59.59	12.82

**Table 4:** Examples of combinations where a certain surface energy component of asphalt was calculated to be zero.

Asphalt Types	Asphalt Surface Energy Component with a Calculated Value of 0	Examples of Reagent Combinations
70# Matrix Asphalt	$\gamma_{s}^{+}$	WFN, WEG, FES, FGN, EGSD, ESBN, SDBN, etc.
	$\gamma_{s}^{-}$	FED, FGB, EBN, GDB, SDN, FEGS, SDBN, etc.
SBS-Modified Asphalt	$\gamma_{S}^{LW}$	WFSB
	$\gamma_{s}^{+}$	WFB, WEB, FED, EGN, WESN, FESB, ESBN, etc.
	$\gamma_{s}^{-}$	FSN, GSB, SDN, FGSD, EGSN, GSBN, SDBN, etc.

**Table 5:** Calculated results of asphalt surface energy parameters with combinations WFSD and WFEG.

Asphalt Type	Calculation Method of Equation	Reagent Combination	Asphalt Surface Energy Parameter/(mJ/m^2^)					$\mathrm{Error}\;\mathrm{Rate}\;\left|\frac{\gamma_{S_{WFEG}}-\gamma_{S_{WFSD}}}{\gamma_{S_{WFEG}}}\right|\times 100\%$
			$\gamma_{S}^{LW}$	$\gamma_{S}^{+}$	$\gamma_{S}^{-}$	$\gamma_{S}^{AB}$	$\gamma_{S}$	
70# Matrix Asphalt	TLS	WFSD	18.28	0.10	1.33	0.73	19.01	17.71%
		WFEG	21.53	0.24	2.57	1.57	23.10	
SBS-Modified Asphalt	TLS	WFSD	18.72	0.21	2.03	1.32	20.04	64.80%
		WFEG	5.99	3.40	2.80	6.17	12.16	

## Data Availability

The original contributions presented in this study are included in the article. Further inquiries can be directed to the corresponding author.

## Appendix A

**Table A1:** Reagent combination and leap degree at every point.

Asphalt Type	Surface Energy Parameter	Leap Degree																				
70# Matrix Asphalt	$\gamma_{S}^{LW}$	WF GB	WF GD	WE GS	WG SD	WF SD	WF DB	WF DN	WG DB	WG DN	WG SB	WF GS	WFD	WSD	FEG	WFE	WF EG	WF ES	FGS	GSD	WFS	WF BN
		1.12	0.67	0.75	0.80	0.83	0.86	0.88	0.89	0.9	0.92	0.94	0.95	0.94	0.93	0.94	0.94	0.95	0.95	0.95	0.98	\
	$\gamma_{s}^{+}$	WF BN	WE GS	WF GD	WF GS	WG SD	WFS	WG SB	WF DB	WF DN	WF SD	FEG	GSD	WSD	WFE	WF EG	WF ES	WFD	WG DB	WG DN	FGS	WF GB
		1.22	1.44	0.93	0.80	0.83	0.99	1.34	0.89	0.90	1.01	1.03	1.07	1.18	0.93	0.94	1.33	1.09	0.95	1.44	1.15	\
	$\gamma_{s}^{-}$	FGS	WF BN	WG DB	WG DN	WF SD	WF DB	WF DN	WG SB	GSD	WF GS	WE GS	WSD	WF GB	FEG	WF GD	WG SD	WFE	WF EG	WF ES	WFS	WFD
		0.93	0.69	0.75	1.85	0.83	0.86	0.89	0.93	0.93	0.93	0.92	0.94	0.96	1.11	0.96	0.96	0.94	0.95	0.98	0.99	\
	$\gamma_{S}$	WF GB	WF GD	WE GS	WG SD	WF SD	WF DB	WF DN	WG DB	WG DN	WG SB	WF GS	FEG	WFD	WSD	WFS	GSD	WFE	WF EG	WF ES	FGS	WF BN
		1.18	0.67	0.75	0.81	0.83	0.86	0.89	0.89	0.90	0.91	0.99	0.92	0.93	0.94	0.94	0.94	0.94	0.95	0.95	0.97	\
SBS-Modified Asphalt	$\gamma_{S}^{LW}$	WF ES	WF GS	WF EG	WG SB	WF GB	WE GS	WF GD	WF SD	WG SD	WG DB	WF DN	WG DN	WF DB	WFD	FEG	WFE	WSD	GSD	WFS	FGS	WF BN
		0.57	0.73	0.78	1.75	0.85	0.97	0.88	0.89	0.90	0.91	0.92	0.92	1.05	0.93	0.94	0.96	0.94	0.95	0.95	0.96	\
	$\gamma_{s}^{+}$	WFS	WSD	GSD	WG SD	WF SD	WF GD	WF BN	WFD	WE GS	WG SB	WFE	FEG	WG DB	WF DN	WG DN	WF DB	WF GB	WF EG	FGS	WF ES	WF GS
		0.50	1.34	1.01	0.81	1.25	1.03	1.15	0.91	0.97	0.99	0.92	1.45	1.41	0.95	0.97	1.09	0.97	0.97	1.06	0.95	\
	$\gamma_{s}^{-}$	WF DB	WG DN	WF DN	WF BN	WG DB	FGS	WF GS	WF GD	WG SB	WE GS	WF SD	WG SD	WFD	WFS	WSD	WF ES	WF GB	WF EG	FEG	WFE	GSD
		0.76	0.93	2.22	1.13	1.21	1.41	1.13	0.97	0.91	0.95	0.92	1.00	1.00	0.93	0.96	0.96	0.95	1.11	0.95	0.97	\
	$\gamma_{S}$	WF GS	WF ES	WF EG	WF GB	WG SB	WE GS	WF DB	WG DN	WF DN	WF SD	WG SD	WF GD	WG DB	WSD	WFS	WFD	GSD	WF BN	FEG	WFE	FGS
		0.53	0.69	0.76	1.08	0.84	0.90	0.88	0.89	0.91	0.91	0.92	0.93	1.04	0.93	0.95	0.94	0.95	0.96	0.95	0.96	\

**Table A2:** The number of outliers.

Reagent Combination	WFE	WFS	WFD	WSD	FEG	FGS	GSD	WF EG	WF ES	WF GS	WF GD	WF GB	WF SD	WF DB	WF DN	WF BN	WE GS	WG SD	WG SB	WG DB	WG DN
Number of Outliers	4	4	3	3	3	4	3	3	4	3	1	4	0	1	1	4	1	1	1	1	2

Annotation: distilled water (W), formamide (F), ethylene glycol (E), glycerol (G), dimethyl sulfoxide (S), diiodomethane (D), benzyl alcohol (B), and n-octanol (N).

## References

[1] (2020). Standard Practice for Effect of Water on Bituminous-Coated Aggregate Using Boiling Water.

[2] Zhang Y., Luo X., Luo R., Lytton R.L. (2014). Crack initiation in asphalt mixtures under external compressive loads. Constr. Build. Mater..

[3] Alfalah A., Ali A., Mehta Y. (2023). Estimating tensile strength ratio of asphalt mixtures using surface free energy (sfe) and fourier transform infrared attenuated total reflectance (ftir-atr). Constr. Build. Mater..

[4] Liu L., Liu L., Yu Y. (2023). Study of factors influencing moisture susceptibility of warm-mix asphalt using the surface free energy approach. Polymers.

[5] Tu C., Xi L., Luo R., Huang T., Luo J. (2022). Effect of water vapor concentration on adhesion between asphalt and aggregate. Mater. Struct..

[6] Little D.N., Bhasin A. (2006). Using Surface Energy Measurements to Select Materials for Asphalt Pavements.

[7] Dong J., Zhang K., Liang S., Ma H., Huang T., Luo R. (2024). Evaluation of moisture sensitivity of asphalt mixtures based on surface energy of asphalt mastic. J. Mater. Civ. Eng..

[8] Mitchell B.S. (2003). An introduction to materials engineering and science: For chemical and materials engineers. Chem. Eng..

[9] Elphingstone G.M. (1997). Adhesion and Cohesion in Asphalt-Aggregate Systems. Ph.D. Thesis.

[10] Cheng D.X. (2002). Surface Free Energy of Asphalt-Aggregate System and Performance Analysis of Asphalt Concrete Based on Surface Free Energy. Ph.D. Thesis.

[11] Bhasin A. (2006). Development of Methods to Quantify Bitumen-Aggregate Adhesion and Loss of Adhesion Due to Water. Ph.D. Thesis.

[12] Tu C., Luo R., Huang T. (2021). Influence of infiltration velocity on the measurement of the surface energy components of asphalt binders using the Wilhelmy plate method. J. Mater. Civ. Eng..

[13] Chibowski E. (2002). Perea-Carpio R. Problems of contact angle and solid surface free energy determination. Adv. Colloid Interface Sci..

[14] Luo R., Zhang D., Zeng Z., Lytton R.L. (2015). Effect of surface tension on the measurement of surface energy components of asphalt binders using the Wilhelmy plate method. Constr. Build. Mater..

[15] Li Q., Xu L., Chen X., Li W., Li Y., Wang H., Liu K. (2024). Study on the adhesion performance of biochar-modified asphalt based on surface free energy and atomic force microscopy. Coatings.

[16] Schuster J.M., Schvezov C.E., Rosenberger M.R. (2015). Analysis of the results of surface free energy measurement of Ti_6_Al_4_V by different methods. Procedia Mater. Sci..

[17] Zhang D., Luo R. (2019). Using the surface free energy (sfe) method to investigate the effects of additives on moisture susceptibility of asphalt mixtures. Int. J. Adhes. Adhes..

[18] Little D.N. (2011). Asphalt Research Consortium Quarterly Technical Progress Report from July to September. Research report.

[19] Al-Rawashdeh A.S., Sargand S. (2014). Performance assessment of a warm asphalt binder in the presence of water by using surface free energy concepts and nanoscale techniques. J. Mater. Civ. Eng..

[20] Singh D., Mishra V. (2018). Different methods of selecting probe liquids to measure the surface free energy of asphalt binders. Constr. Build. Mater..

[21] Hefer A.W., Bhasin A., Little D.N. (2006). Bitumen surface energy characterization using a contact angle approach. J. Mater. Civ. Eng..

[22] Kwok D.Y., Lin R., Mui M., Neumann A.W. (1996). Low-rate dynamic and static contact angles and the determination of solid surface tensions. Colloids Surf. A: Physicochem. Eng. Asp..

[23] Ma F., Fu Z., Wang L.L. (2014). Asphalt-aggregate adhesion work of natural asphalt modified asphalt. Int. J. Pavement Res. Technol..

[24] (2011). Standard Test Methods of Bitumen and Bituminous Mixtures for Highway Engineering.

[25] (2017). Standard Test Methods for Surface and Interfacial Tension of Solutions of Paints, Solvents, Solutions of Surface-active Agents, and Related Materials.

[26] Kurt O., Arslan O. (2012). Geometric interpretation and precision analysis of algebraic ellipse fitting using least squares method. Acta Geod. Et Geophys. Hung..

[27] Marquardt D.W. (1963). An algorithm for least-squares estimation of nonlinear parameters. J. Soc. Ind. Appl. Math..

[28] Markovsky I., Van Huffel S. (2007). Overview of total least-squares methods. Signal Process..

[29] Gu T., Lin S., Fang B., Luo T. (2016). An improved total least square calibration method for straightness error of coordinate measuring machine. Proc. Inst. Mech. Eng. Part B J. Eng. Manuf..

[30] Ord K. (1996). Outliers in statistical data. Int. J. Forecast..

[31] Arthur Z., Peter F. (2018). There and back again: Outlier detection between statistical reasoning and data mining algorithms. Wiley Interdiscip. Rev. Data Min. Knowl. Discov..

[32] Onoz B., Oguz B. (2003). Assessment of Outliers in Statistical Data Analysi.

[33] Wang X., Zeng R., Zou F., Huang F., Jin B. (2021). A highly efficient framework for outlier detection in urban traffic flow. IET Intell. Transp. Syst..

